# Inter- and Intraobserver variability of attenuation coefficient measurement in innovative ultrasound diagnosis of metabolic dysfunction-associated steatotic liver disease: a cross-sectional study

**DOI:** 10.3389/fmed.2025.1457960

**Published:** 2025-03-13

**Authors:** Maksym Zhaivoronok, Oleh Dynnyk, Olexandr Livkutnyk, Viktoriia Yerokhovych, Violetta Yuzvenko, Iryna Serednia, Yelizaveta Melnychenko, Nazarii Kobyliak

**Affiliations:** ^1^Department of Nuclear Medicine, Radiation Oncology and Radiation Safety of Shupyk National Healthcare University of Ukraine, Kyiv, Ukraine; ^2^“Institute of Elastography” Medical Center LLC, Kyiv, Ukraine; ^3^Kyiv City Clinical Endocrinology Center, Kyiv, Ukraine; ^4^Endocrinology Department, Bogomolets National Medical University, Kyiv, Ukraine; ^5^Ukrainian Scientific and Practical Center for Endocrine Surgery, Transplantation of Endocrine Organs and Tissues of the Ministry of Health of Ukraine, Kyiv, Ukraine; ^6^Medical Laboratory CSD, Kyiv, Ukraine

**Keywords:** interobserver and intraobserver variability, attenuation coefficient, steatometry, steatotic liver disease, metabolic dysfunction-associated steatotic liver disease

## Abstract

**Introduction:**

Evaluation of the ultrasound attenuation coefficient is widely used in the diagnosis of steatotic liver disease (SLD). US steatometry with real-time attenuation coefficient measurement (ACM) is an imaging tool that can replace and surpass the B-mode and improve the noninvasive diagnosis of SLD.

**Aim:**

To evaluate the intra- and interobserver variability of ACM for the assessment of SLD.

**Materials and methods:**

A single-center cross-sectional study was conducted at the Kyiv City Clinical Endocrinology Center. We examined 52 patients (25 men and 27 women) with a mean age of 53.2 ± 4.73 years. B-mode and ACM were performed on a Soneus P7 US system (Ultrasign, Ukraine). Examinations were performed by 2 radiologists with 28 (expert 1) and 17 (expert 2) years of experience and 4 general practitioners (GPs) without US experience (nonexperts 1–4). The training of 4 GPs on mastering the ACM was only 60 min due to US steatophantom. Each doctor performed 5 measurements of the ACM for each patient. The inter- and intraobserver variability of the results was determined by using an intraclass correlation coefficient (ICC) with a 95% confidence interval (95% CI).

**Results:**

The overall intraobserver variability after 5 days of examination was as follows: for expert 1–0.958 (95% CI 0.938–0.974); for expert 2–0.936 (95% CI 0.905–0.980); nonexpert 1–0.891 (95% CI 0.843–0.929); nonexpert 2–0.915 (95% CI 0.876–0.945); nonexpert 3–0.927 (95% CI 0.893–0.953); nonexpert 4–0.880 (95% CI 0.827–0.927). Interobserver variability at the final timepoint (day 5) was as follows: between experts 1 and 2, 0.942 (95% CI 0.898–0.967); between nonexperts 1–4 overall, 0.871 (95% CI 0.800–0.921); and overall, 0.922 (95% CI 0.883–0.951).

**Conclusion:**

Real-time US steatometry with ACM measurement is an informative, simple method with excellent intra- and interobserver variability and a reproducible method for population assessment for the early diagnosis and staging of SLD. The simplicity of ACM technology allows general practitioners to master the technique within 60 min. ACM measurements can be effectively employed by general practitioners (GPs) for population screening, enabling timely identification and management of MASLD.

## Introduction

Metabolic dysfunction–associated steatotic liver disease (MASLD) is the most common pathology among all chronic diffuse diseases of this organ and leads to a deterioration in quality of life, disability and mortality in large regions of the population (liver-related morbidity and mortality) (1, 2). This is due to the high risk of progression of MASLD to the development of nonalcoholic steatohepatitis (NASH), cirrhosis, hepatic failure and hepatocellular carcinoma (3). The prevalence of MASLD in different countries reaches 20–40% (4–6). Due to the significant global problem of MASLD, there is a significant need to find an optimal and cost-effective diagnostic solution for the population assessment of steatotic liver disease (SLD) (7, 8).

SLD is quite common in patients who undergo conventional ultrasound (US) of the abdominal organs on a B-mode grayscale, especially in patients with pathological liver tests. In addition, SLD detection should prompt clinicians to seek an association with diabetes mellitus (DM), hypertension, hypertriglyceridaemia, and low high-density lipoprotein (HDL) cholesterol (9). Hence, the presence of components of metabolic syndrome is a prerequisite for US detection of SLD (10).

However, the US grayscale B-mode for SLDs is based on a subjective assessment of liver echogenicity, hepatorenal echo contrast, bright liver, and deep attenuation; thus, it is a rather operator-dependent technique (11). There are known studies of inter- and intraobserver variability in the assessment of SLD in the grayscale B-mode in general clinical practice (12).

The main innovation in US of the SLD is the measurement of the attenuation coefficient (AC), which is more accurate and objective and allows the quantification and grading of steatosis (13, 14). The controlled attenuation parameter (CAP™, FibroScan) is the first accurate US method available for quantifying hepatic steatosis worldwide, and it has become useful for point-of-care US (POCUS) (15–17). Using AC, modern US devices allow accurate navigation of the region of interest (ROI), avoid artifacts (reverberations, shadows, etc.), and exclude the measurement of undesirable anatomical structures in the ROI (18). These features of ultrasound devices can contribute to better AC performance than CAP performance and reproducibility of measurements (16). The creation of a hand-held US device (HHUSD) with AC technology opens the prospect of its widespread use in population studies of MASLD and equipment for POCUS by general practitioners.

To reliably assess the SLD, AC should be a reproducible technique among investigators and for the same operator. Currently, no study has described the reproducibility of real-time US steatometry with AC in a cohort of SLD patients. US steatometry with real-time attenuation coefficient measurement (ACM) is an imaging tool that can replace and surpass the B-mode and improve the noninvasive diagnosis of SLD.

This work aimed to evaluate the intra- and interobserver variability of attenuation coefficient measurements (ACMs) for the assessment of the SLD.

## Materials and methods

### Ethics statement

The primary research protocol was approved by the local Ethics Committee of Shupyk National Healthcare University of Ukraine and put into practice on the basis of the Declaration of Helsinki (1975). The present study was conducted in compliance with the principles of secrecy and confidentiality. The collection and analysis of the patients’ information were performed anonymously, and a code was used to prevent disclosure. Patients were free to leave the study at any time during the study, without the need to provide any explanation or reason.

### Study design

A single-center cross-sectional study was conducted at Kyiv City Clinical Endocrinology Center and included 52 patients with different endocrine pathologies. All patients were examined using a single diagnostic algorithm, which included several stages: (1) patient questionnaires (identification of complaints indicating the presence of liver disease, concomitant pathology, arterial hypertension, type 1 and type 2 DM, burdened family history and alcohol abuse) and anthropometry; (2) real-time US steatometry of patients using ACM technology by six independent researchers; and (3) intra- and interobserver comparisons of results obtained with ultrasound. Patients with a heterogeneous echo pattern of the liver parenchyma in B-mode or ascites were excluded from the study. Patients underwent abdominal US for indications not necessarily related to liver pathology. The images were viewed by observers on the same monitor and under the same ambient lighting conditions.

Exclusion сriteria: patients who exhibited heterogeneous echo patterns of the liver parenchyma, such as the “geographic” form of liver steatosis or regenerated nodules in cirrhosis, as well as those with ascites, were excluded from the study. These criteria were applied to ensure the accuracy and consistency of ultrasound imaging interpretation, as such conditions could confound the assessment of hepatic steatosis.

All the patients underwent anthropometry, and the following data were collected: body height (BH) accurate to 0.001 m and body weight (BW) accurate to 0.001 kg using medical scales. The Quetelet formula was used to calculate the individual’s body mass index (BMI):

BMI = BW/BH^2^.

Waist circumference (WC) was measured using a flexible tape at the belly button level accurate to 0.001 m.

### US steatometry

Measurements were performed in B-mode and by the ACM method in the liver parenchyma on a Soneus P7 ultrasound device (Ultrasign, Ukraine) with a C1.5–5.0 MHz convex sensor. This study involved 2 radiologists with 28 (expert 1) and 17 (expert 2) years of experience and 4 general practitioners (GPs) without experience in the use of ultrasound devices (nonexpert 1–4).

**Figure 1:**
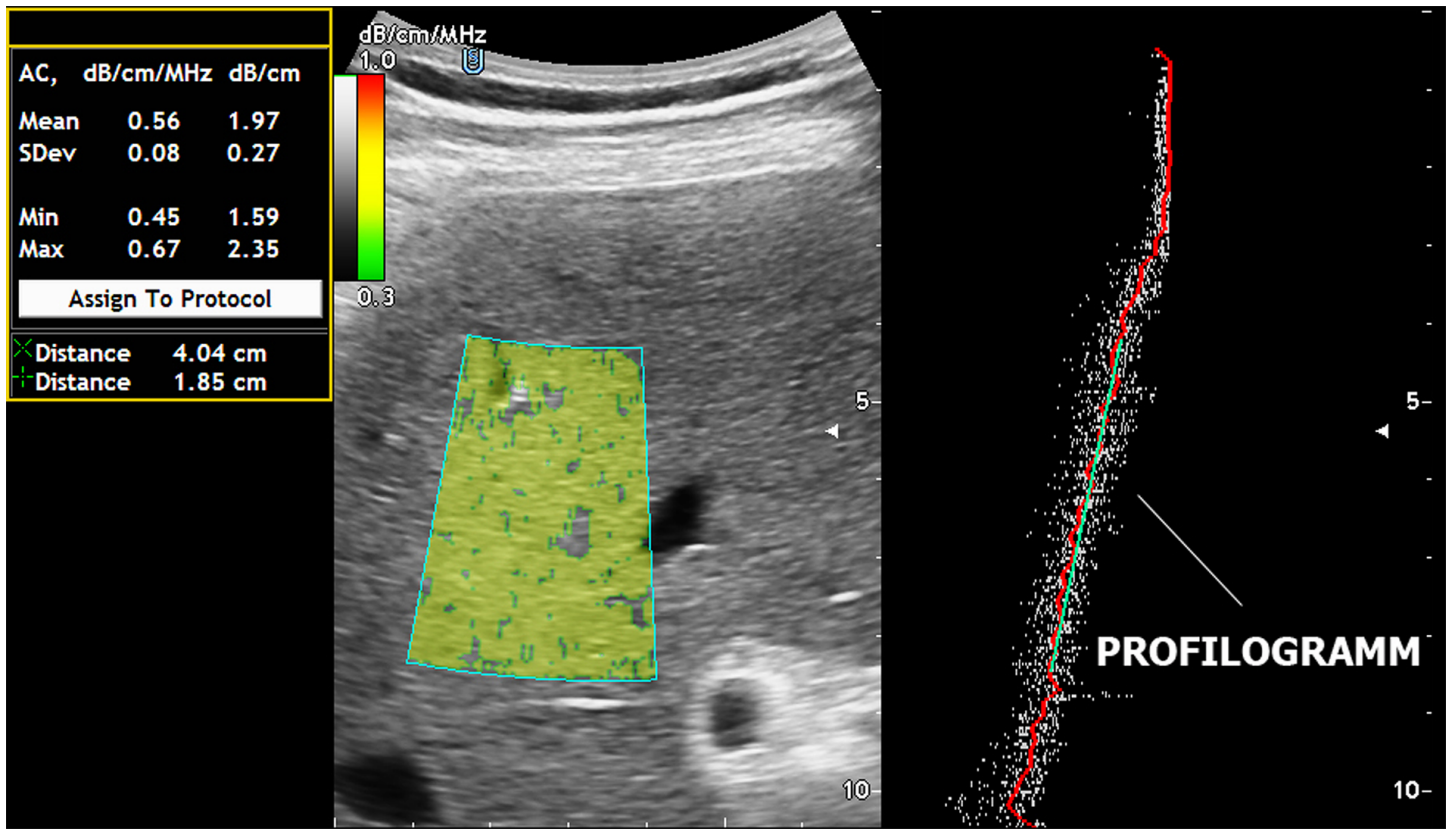
Real-time US steatometry using the ACM method. The profilogram of US wave attenuation is employed for easy navigation within the area of interest and for quality control of the obtained data.

The training of 4 GPs for ACM mastering took 60 min with the use of US steatophantoms (18). Real-time ACM was performed one at a time for each patient by each of the 6 operators separately without the presence of other operators to prevent peers from becoming aware of the ACM results. Each investigator performed 5 ACM measurements for each patient and recorded the obtained values in dB/cm. The patients were examined while they were not breathing. US was performed using the “free hand” technique, and the sensor was located parallel to the rib arch in the intercostal space to select the optimal “acoustic window” for the ROI of the ACM. Each operator independently carried out the ROI for the ACM in the liver parenchyma, avoiding the inclusion of large vessels (>10 mm in diameter), bile ducts and liver capsules. The ROIs were always positioned 15–20 mm deeper than the liver capsule, bypassing reverberations. The optimal depth of the ROI was determined from the linear segment of the attenuation graph (profilogram). The AMC profilogram made it easy to avoid ultrasound artifacts even for US steatometry beginners (Figure 1).

To minimize intra- and interobserver variability, we implemented training protocols for all participating sonographers and employed standardized imaging protocols. Additionally, multiple independent evaluations were conducted to ensure consistency in measurements.

The distribution of SLDs according to the steatometry results was determined according to the US attenuation scale proposed by Sasso et al. (19): the S0 stage corresponds to the norm (the fraction of hepatocytes with fat ranges from 0 to 5%), from 1.0 to 2.19 dB/m/MHz; the S1 stage corresponds to the mild stage (6–33%), from 2.20 to 2.29 dB/m/MHz; the S2 stage corresponds to the moderate stage (34–66%), from 2.30 to 2.90 dB/m/MHz; and the S3 stage corresponds to the severe stage (> 66%), > 2.90 dB/m/MHz (19).

### Statistical analysis

Statistical analysis was performed using the standard software SPSS version 20.0 (SPSS, Inc., Chicago, Illinois) and GraphPad Prism, version 6.0 (GraphPad Software, Inc., La Jolla, CA, USA). Quantitative variables are presented as the mean and standard deviation of the mean (M ± SD), and qualitative variables are presented as %. The ACM is represented graphically in the form of a box chart. The upper and lower bars correspond to the 25^th^ and 75^th^ quartiles, respectively, and the line passing through the middle of the square corresponds to the median value (Me). To test the normality of the distribution, the Kolmogorov–Smirnov one-sample test was used. Continuous variables with a parametric distribution were analysed using analysis of variance (ANOVA), and if the results were significant, a *post hoc* Tukey’s test was performed. For nonnormally distributed data, the Kruskal–Wallis test was used for multiple comparisons. For comparisons of categorical variables, we conducted a χ^2^ test. Differences between the compared groups were considered statistically significant at *p* < 0.05. Significant differences between the groups are marked using the letters *a* and *b*.

The inter- and intraobserver variability of the results was determined by using an intraclass correlation coefficient (ICC) with a 95% confidence interval (95% CI). An ICC less than 0.5 indicates low reliability, an ICC from 0.5 to 0.75 indicates moderate reliability, an ICC from 0.75 to 0.9 indicates good reliability, and an ICC greater than 0.90 indicates excellent reliability. The endpoints of the study were achievements by all investigators with an ICC greater than 0.9 on one day of examination as part of personal (intraobserver) and generalized interobserver agreement.

## Results

We examined 52 patients (25 men and 27 women) with a mean age of 53.2 ± 4.73 years. Among them, 6 were reasonably healthy (11.5%), 26 had type 2 DM (50%), 8 had type 1 DM (15.4%), 6 had obesity (11.5%), 4 had autoimmune thyroiditis (7.7%), and 1 each had Graves’ disease and acromegaly (1.9%) (Table 1).

**Table 1:** Baseline clinical parameters of the examined patients (mean ± SD or %).

Parameters	Value
Age, years	53.2 ± 4.73
reasonably healthy, % (*n*)	11.5 (6)
Type 2 DM, % (*n*)	50 (26)
Type 1 DM, % (*n*)	15.4 (8)
Obesity, % (*n*)	11.5 (6)
Autoimmune thyroiditis, % (*n*)	7.7 (4)
Graves’ disease, % (*n*)	1.9 (1)
Acromegaly, % (*n*)	1.9 (1)
Diabetes duration, years	11.92 ± 9.48
Weight, kg	86.92 ± 16.89
BMI, kg/m^2^	30.59 ± 6.20
Waist circumference, cm	101.75 ± 14.51

The overall intraobserver variability after 5 days of examination was as follows: for expert 1–0.958 (95% CI 0.938–0.974); for expert 2–0.936 (95% CI 0.905–0.980); nonexpert 1–0.891 (95% CI 0.843–0.929); nonexpert 2–0.915 (95% CI 0.876–0.945); nonexpert 3–0.927 (95% CI 0.893–0.953); nonexpert 4–0.880 (95% CI 0.827–0.927) (Table 2). Notably, expert 1 reached the endpoint of the study (ICC > 0.9) on day one, expert 2 on day two, and all GPs reached the endpoint by day 4 (Table 2).

**Table 2:** Intraobserver variability for ACM measurement in examined patients (ICC; 95% CI).

	Day 1	Day 2	Day 3	Day 4	Day 5	Overall
**Expert 1**	**0.920** (0.747; 0.990)	**0.938** (0.877; 0.976)	**0.957** (0.899; 0.987)	**0.964** (0.917; 0.989)	**0.987** (0.969; 0.996)	**0.958** (0.938; 0.974)
**Expert 2**	**0.893** (0.677; 0.987)	**0.917** (0.838; 0.967)	**0.963** (0.913; 0.989)	**0.976** (0.944; 0.993)	**0.979** (0.949; 0.994)	**0.936** (0.905; 0.980)
**Nonexpert 1**	**0.761** (0.415; 0.967)	**0.837** (0.701; 0.932)	**0.947** (0.877; 0.984)	**0.937** (0.861; 0.980)	**0.932** (0.845; 0.980)	**0.891** (0.843; 0.929)
**Nonexpert 2**	**0.729** (0.365; 0.961)	**0.880** (0.772; 0.951)	**0.940** (0.862; 0.982)	**0.967** (0.925; 0.990)	**0.969** (0.925; 0.991)	**0.915** (0.876; 0.945)
**Nonexpert 3**	**0.840** (0.576; 0.979)	**0.961** (0.921; 0.985)	**0.914** (0.808; 0.974)	**0.924** (0.835; 0.976)	**0.960** (0.906; 0.988)	**0.927** (0.893; 0.953)
**Nonexpert 4**	**0.723** (0.374; 0.960)	**0.849** (0.721; 0.938)	**0.861** (0.706; 0.957)	**0.940** (0.867; 0.981)	**0.924** (0.829; 0.977)	**0.880** (0.827; 0.927)

The bold indicates significant changes.

The endpoint for overall interobserver variability was reached on day 4 of the study, which was also confirmed on day 5. At the end of the study, the ICCs for interobserver agreement were as follows: between experts 1 and 2, 0.942 (95% CI 0.898–0.967); between nonexperts 1–4 overall, 0.871 (95% CI 0.800–0.921); and overall, 0.922 (95% CI 0.883–0.951) (Table 3).

**Table 3:** Interobserver variability for ACM measurement in examined patients (ICC; 95% CI).

	Experts	Nonexperts	Overall
**Day 1**	**0.875** (0.717; 0.945)	**0.751** (0.541; 0.879)	**0.717** (0.501; 0.859)
**Day 2**	**0.915** (0.762; 0.898)	**0.774** (0.699; 0.838)	**0.784** (0.719; 0.843)
**Day 3**	**0.920** (0.859; 0.955)	**0.890** (0.830; 0.932)	**0.622** (0.511; 0.732)
**Day 4**	**0.850** (0.742; 0.912)	**0.843** (0.762; 0.901)	**0.906** (0.861; 0.940)
**Day 5**	**0.942** (0.898; 0.967)	**0.871** (0.800; 0.921)	**0.922** (0.883; 0.951)

The bold indicates significant changes.

**Figure 2:**
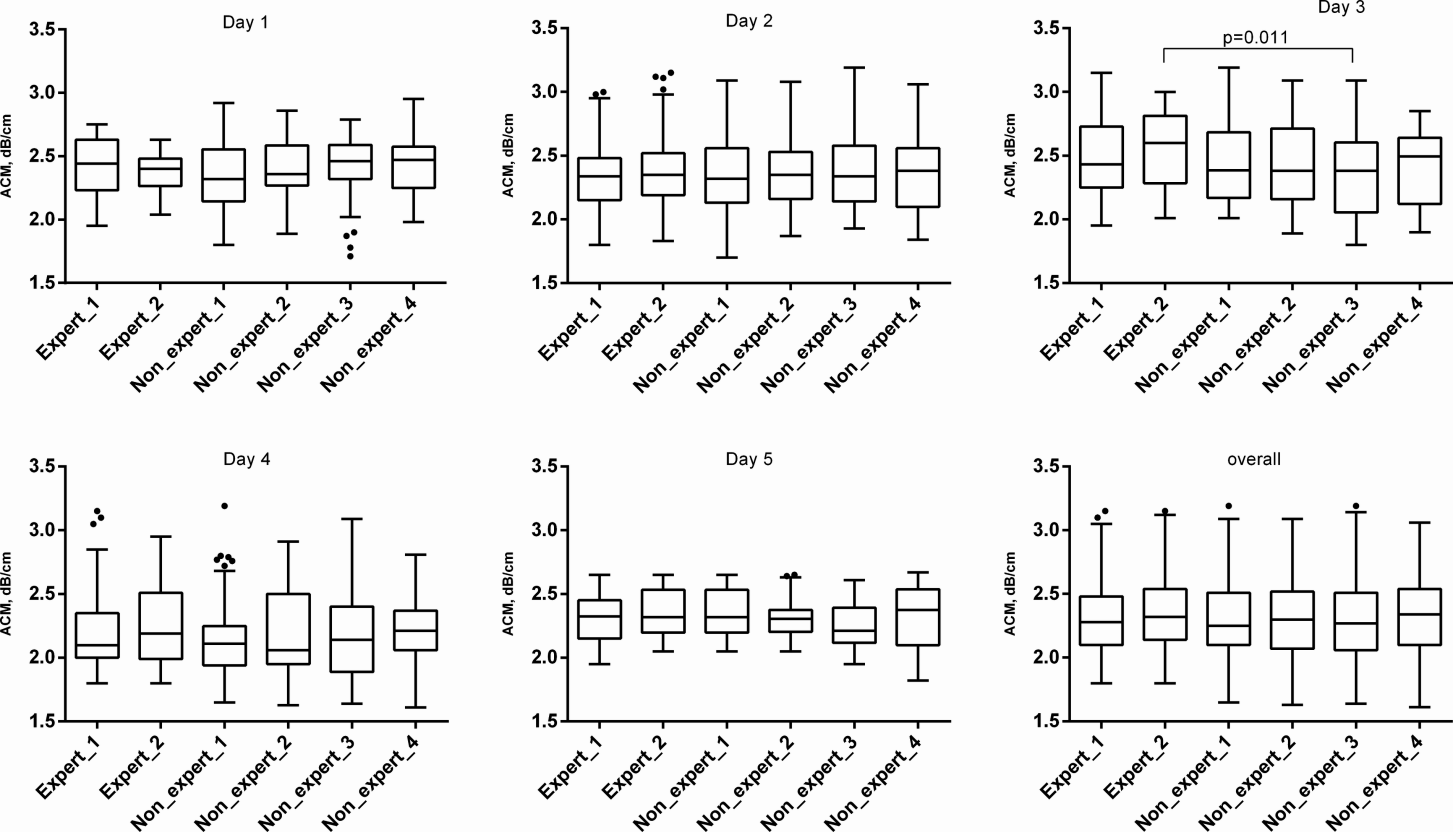
ACM values in examined patients. The ACM is represented graphically in the form of a box chart. The upper and lower bars correspond to the 25th and 75th quartiles, respectively, and the line passing through the middle of the square corresponds to the median value (Me).

The most difficulty in terms of the discrepancy of the results was the 3^rd^ day, when the interobserver variability of the results differed both between the experts themselves and between the experts and the GPs (Table 3). On the same day, significantly different results were noted for the mean ACM between expert 1 and nonexpert 3 (*p* = 0.011) (Table 4; Figure 2). On this day, in 3 patients, all operators, without exception, encountered the same difficulties in imaging the liver due to a poor-quality “acoustic window” with standard intercostal access in both B-mode and ACM. In one type 2 DM patient, this was associated with acoustically dense subcutaneous fat (hypodermis), which was consistent with the use of echo semiotics for chronic cellulitis. In another patient with type 2 DM, acoustic access was difficult due to peritonitis during laparoscopic cholecystectomy. This may have been a manifestation of the unification of the liver capsule with the parietal leaf of the peritoneum and the fixation between them of gas residues from the laparoscopic pneumoperitoneum. In a patient with acromegaly, the intercostal spaces were narrowed with excessive calcification of the ribs, which narrowed the “acoustic window.” Such conditions can explain part of unsuccessful US steatometry measurements and can be alternatively resolved by another radiological modality, magnetic resonance proton density fat fraction (MRI-PDFF).

**Table 4:** ACM values in examined patients.

	Day 1	Day 2	Day 3	Day 4	Day 5	Overall
Expert 1	2.36 ± 0.16	2.38 ± 0.29	2.57 ± 0.28^b^	2.11 ± 0.22	2.35 ± 0.18	2.35 ± 0.28
Expert 2	2.41 ± 0.21	2.32 ± 0.25	2.47 ± 0.29^ab^	2.08 ± 0.21	2.29 ± 0.17	2.30 ± 0.27
Nonexpert 1	2.35 ± 0.32	2.35 ± 0.3	2.42 ± 0.29^ab^	2.02 ± 0.22	2.31 ± 0.18	2.29 ± 0.30
Nonexpert 2	2.37 ± 0.25	2.37 ± 0.26	2.41 ± 0.32 ^ab^	2.06 ± 0.29	2.31 ± 0.15	2.30 ± 0.29
Nonexpert 3	2.38 ± 0.30	2.40 ± 0.32	2.36 ± 0.32^a^	2.07 ± 0.26	2.25 ± 0.19	2.29 ± 0.31
Nonexpert 4	2.44 ± 0.23	2.37 ± 0.31	2.41 ± 0.28^ab^	2.12 ± 0.24	2.32 ± 0.25	2.32 ± 0.29
	*p* = 0.824	*p* = 0.667	*p* = 0.021	*p* = 0.299	*p* = 0.209	*p* = 0.129

The data are presented as the mean ± SD. One-way ANOVA with post hoc Tukey’s test for multiple comparisons was performed for data analysis. a, b Values in the same column with different superscript letters show significant differences at *p* < 0.05.

It is also worth noting that there was no significant difference between experts and GPs regarding the diagnosis and grading of SLD, which indicates the sensitivity of the innovative ACM technique and the possibility of using it for population screening of early forms of steatosis (Table 5).

**Table 5:** Prevalence and distribution by SLD degree.

	Expert 1	Expert 2	Nonexpert 1	Nonexpert 2	Nonexpert 3	Nonexpert 4	p
S0, % (*n*)	33.3 (17)	37.3 (19)	41.2 (21)	35.3 (18)	41.2 (21)	37.3 (19)	0.956
S1, % (*n*)	17.6 (9)	13.7 (7)	15.6 (8)	13.7 (7)	9.8 (5)	7.8 (4)	0.701
S2, % (*n*)	43.2 (22)	45.1 (23)	41.2 (21)	49.0 (25)	45.1 (23)	52.9 (27)	0.869
S3, % (*n*)	5.9 (3)	3.9 (2)	2.0 (1)	2.0 (1)	3.9 (2)	2.0 (1)	0.903

## Discussion

SLD is increasingly recognized as a serious clinical problem and is now considered the most common liver disease in developed countries (20). The issue of US is relevant when referring patients for examination to assess SLDs and should be diagnosed on a daily basis in the routine practice of different radiological and other (gastro/hepatology) departments with different equipment and operator experience. To be clinically useful in SLD assessment according to the ACM method, US steatometry must have reliability and reproducibility between investigators. Additionally, different researchers can measure the ACM differently for the same patient because the navigation of the ROI from the main vendors of US equipment is arbitrarily selected by the operator. There are no previous studies in the literature that have assessed the actual variability among ACM implementers in real time. Previously, ultrasound SLD evaluation was highly operator dependent, and ultrasound conclusions were based primarily on subjective assessment of liver echogenicity (13). The echogenicity of the liver is normally equal to or slightly greater than the echogenicity of the kidney cortex, but this criterion is highly dependent on the visual perception of a researcher. In addition, the echogenicity of the right kidney cortex can be altered by a pathological process in the kidney itself (21). Due to the way the ACM ROI is navigated by the attenuation profile in the device used, it is easy for an inexperienced operator to obtain fairly accurate US results. A well-defined and simple algorithm for the operator’s actions when scanning the liver through intercostal spaces in real time in B-mode is aimed only at obtaining an image of the liver area up to 2–3 cm wide and in the anterior–posterior direction up to 4 cm for the location of the ACM ROI. An important methodological point is to obtain information about the ACM from a segment of the profilogram graph exclusively with a linear profile of US wave attenuation. This greatly simplifies and speeds up the execution of the entire procedure from the set of 5 ACM measurements. The ACM profilogram easily allows the operator to avoid acoustic artefacts, such as shadows from the ribs and air in the lung sinus and reverberations from the layers of the body preceding the liver (dermis, hypodermis, fascia, muscles, leaf of the parietal peritoneum and capsules of the liver). The profilogram also allows the operator not to be exposed to the large portal tracts, diaphragm, capsule and portal of the liver, as well as to bypass the gallbladder and gas in the intestine.

The variability in the interpretability of radiological images of patients with SLDs was first reported by Saadeh et al. (22). Their study included an assessment of intra- and interobserver agreement on the nature and severity of the disease using B-mode US, multidetector computed tomography (MDCT), and magnetic resonance imaging (MRI). The intraobserver agreement for US on the severity of steatosis was substantial (0.63), but the agreement between observers on the severity of steatosis was satisfactory (0.40). Our study differed from Saadeh et al. in several aspects. Their study enrolled a preselected cohort of 25 patients with clinical and laboratory data from MASLD, while our study population included 52 patients who were appointed to the medical center without a preestablished diagnosis. On the other hand, Saadeh et al. showed that the severity of steatosis was only accurately determined when liver biopsy detected more than 33% of hepatocytes. Agreement levels were also expected to be higher in our study if they were limited to patients with S2-S3 (23).

The findings from our study indicate that ACM reduces the operator dependency that has long been a challenge in US-based SLD assessments. By standardizing the process of ROI navigation and minimizing the influence of common US artifacts, ACM ensures more reliable results even when conducted by less experienced operators. This is particularly important in routine clinical settings where operator skill levels may vary.

A study by Cengiz M et al. demonstrated that the visual assessment of hepatic steatosis using ultrasound has significant interobserver variability and limited reproducibility (24). Comparing our findings to those of Saadeh et al., who reported substantial intraobserver variability and only moderate interobserver agreement in SLD assessment using B-mode US, CT, and MRI (23), highlights the improved reliability of ACM. Jeon SK et al. in similarly, studies utilizing CAP have demonstrated variability in liver fat quantification (25).

An important methodological aspect is to derive relevant information about the optimal ACM ROI position exclusively from a segment of the profilogram graph with a linear profile of ultrasound wave attenuation.

Our study also underscores the rapid adaptability of ACM among GPs, with a learning curve plateauing by the second day of training. This suggests that ACM can be seamlessly incorporated into routine practice, facilitating broader population screening for SLD. The detection of mild steatosis (S1) in a significant proportion of cases (33–41.2%) across varying levels of operator experience further supports ACM’s sensitivity and its potential role in early disease detection. Early identification is crucial in managing MASLD to prevent its progression to more severe liver conditions.

The clinical implications of these findings are substantial. By enabling accurate SLD diagnosis and staging in primary care settings, ACM has the potential to transform the management of MASLD, allowing for timely interventions that could improve patient outcomes and reduce the overall healthcare burden associated with advanced liver disease.

Despite these promising results, our study’s limitations, including its single-center design and relatively small sample size, must be acknowledged. To enhance the generalizability of our findings, future studies should involve larger, more diverse populations and multiple centers. This would further validate ACM’s effectiveness and establish its role in the broader clinical management of MASLD.

In conclusion, our study contributes significantly to the development of early diagnostic methods for MASLD. ACM represents a promising advancement in the noninvasive assessment of SLD, offering a reliable, reproducible, and operator-independent diagnostic tool that can enhance early detection and screening, ultimately improving patient care and outcomes in the context of MASLD.

## Conclusion

Real-time US steatometry with ACM measurement is an informative, simple and reproducible method for population assessment for the early diagnosis and staging of SLD. ACM measurements can be used at the primary level by GPs (as an example of an operator without significant US experience) for screening early forms of SLD to overcome the MASLD pandemic and its catastrophic consequences.

## Data Availability

The raw data supporting the conclusions of this article will be made available by the authors, without undue reservation.
